# *CLU* Polymorphisms in Patients with Pseudoexfoliation Syndrome in Polish Population

**DOI:** 10.1155/2019/8787149

**Published:** 2019-07-01

**Authors:** Hanna Lesiewska, Katarzyna Linkowska, Joanna Stafiej, Tomasz Grzybowski, Jacek Swobodziński, Grażyna Malukiewicz

**Affiliations:** ^1^Department of Ophthalmology, The Nicolaus Copernicus University, Ludwik Rydygier's Collegium Medicum in Bydgoszcz, Skłodowskiej Curie 9, 85-094 Bydgoszcz, Poland; ^2^Department of Molecular and Forensic Genetics, Institute of Forensic Medicine, The Nicolaus Copernicus University, Ludwik Rydygier's Collegium Medicum Ludwik Rydygier, Skłodowskiej Curie 9, 85-094 Bydgoszcz, Poland

## Abstract

**Purpose:**

To evaluate *CLU* polymorphisms in patients with pseudoexfoliation syndrome.

**Materials and Methods:**

We studied 81 patients (23 males and 58 females, the median age 76 years) and 91 control subjects (27 males and 64 females, the median age 75 years). Genotypes of the *CLU* polymorphisms (SNPs), rs3087554 and rs2279590, were determined using a commercially available validated genotyping assays. The *χ*^2^ test was performed to compare patient and control groups for possible associations between SNP genotype/allele frequency and disease state.

**Results:**

There were no significant differences for both allele and genotype frequencies between PEX patients and controls for rs3087554 and rs2279590 polymorphisms. The haplotypes distribution shows statistically significant difference between groups (*p*=0.03). The haplotype (CT) more often was found in controls than in PEX patients, conferring an 18-fold decreased risk to the disease.

**Conclusion:**

Our results indicate that CLU variants may contribute to the risk of PEX in the Polish population.

## 1. Introduction

Pseudoexfoliation syndrome (PEX) is a form of age-related elastosis resulting from the overproduction or overaggregation of elastic microfibrillar components [1]. There is an increasing prevalence of PEX as the mean age of the general population increases. The role of inheritance in PEX is still unclear. Several studies demonstrated an increased prevalence of pseudoexfoliation in relatives of family members affected with pseudoexfoliation compared to the general population [2–5]. These findings encouraged other investigators to identify possible genetic factors which could be involved. The genome-wide study conducted in populations of Iceland and Sweden showed the strong association between two single-nucleotide polymorphisms (SNPs) in the lysyl oxidase-like gene and PEX syndrome [6]. This association was then confirmed in many populations worldwide, including our present group of PEX patients [7–13]. The gene expression analyses revealed several genes that may play a role in PEX syndrome pathology, where clusterin was one among them (CLU) [14]. As the results of the studies on CLU association with PEX syndrome were inconclusive, we decided to determine this possible association in the Polish population.

## 2. Materials and Methods

We studied 81 patients (23 males and 58 females, the median age 76 years, *Q*_1_ = 72.0; *Q*_3_ = 82.0) and 91 control subjects (27 males and 64 females, the median age 75 years, *Q*_1_ = 70.0; *Q*_3_ = 80.0), who presented to the Department of Ophthalmology, Collegium Medicum UMK in Bydgoszcz, Poland, for cataract surgery. This work has been approved by the local bioethical committee. All patients gave their informed consent for this study. Patients were enrolled into the study if they had no other ocular or general diseases e.g., glaucoma, age-related macular degeneration (AMD), diabetes, dislipidemia, and arterial hypertension, except cataract and PEX. Glaucoma was defined based on measurements of IOP consistently >21 mmHg without glaucoma medication and the presence of typical glaucomatous optic nerve and visual field changes or previously diagnosed glaucoma under treatment. AMD was diagnosed on the basis of the presence of hard and soft drusen, changes in the retinal pigment epithelium (RPE), geographic atrophy, choroidal neovascular membrane, or disciform scar. In every patient, the diagnosis of PEX was confirmed by slit-lamp examination after pupil dilation. Pseudoexfoliation changes were identified as the presence of typical PEX material on the anterior lens surface, iris, or corneal endothelium. The individuals without any evidence of pseudoexfoliation deposits on intraocular tissues were taken as the control group.

### 2.1. Genotyping

DNA extracts obtained from patients' blood by using the Gene Matrix Bio-Trace DNA Purification Kit (Eurx Ltd., Gdańsk) as part of previous PEX research were reused. The DNA concentration was measured using a spectrophotometer (DeNovix). Genotypes of the *CLU* SNPs, rs3087554, and rs2279590 were determined using a commercially available validated genotyping assay, TaqMan SNP genotyping assay (assay ID: C___1187215_10 i C___1842470_20) (Applied Biosystems) with the ViiA™ 7 real-time PCR system (Applied Biosystems) in accordance with the manufacturer's instructions. The DNA concentration in PCR reaction was 1 ng/*μ*l in total volume 10 *μ*l. Due to the small amount of the material, we were not able to determine both SNPs in all patients.

### 2.2. Statistical Analysis

The *χ*^2^ test was performed to compare patient and control groups for possible associations between SNP genotype/allele frequency and disease state. The Arlequin software version 3.1 was used to determine the Hardy–Weinberg equilibrium and to estimate haplotype frequencies. Odds ratios were also calculated. The significance level for all statistical tests was 0.05. Statistical analysis was performed using Statistica software (version 12).

## 3. Results

Two SNPs of *CLU* were assessed: rs3087554 in 81 PEX patients and 91 controls and rs2279590 in 67 PEX patients and 50 controls. Due to the small amount of material, both SNPs were determined only in some probes. Allelic frequencies of SNPs rs3087554 and rs2279590 were in the Hardy–Weinberg equilibrium in both groups.

Allele and genotype frequencies of rs3087554 polymorphism are presented in Tables 1 and 2. There were no significant differences for both allele and genotype frequencies between PEX patients and controls; *p*=0.94 and *p*=0.83, respectively.

Allele and genotype frequencies of rs2279590 polymorphism are presented in Tables 3 and 4. Also for this polymorphism, there were no statistically significant differences in the frequency of alleles and genotypes between the groups; *p*=0.54 and *p*=0.41, respectively.

The frequencies of *CLU* haplotypes are presented in Table 5.

The haplotypes distribution shows statistically significant difference between groups (*p*=0.03). The haplotype (CT) more often was found in controls than in PEX patients (*p*=0.0484), conferring an 18-fold decreased risk to the disease. The risk of patients with haplotype (CT) for developing PEX is 0.0545. The haplotype (TT) nearly doubles the risk of pseudoexfoliation, but this result was not statistically significant (*p*=0.0687).

## 4. Discussion

Several lines of evidence, including regional clustering, transmission in two-generation families, familial aggregation, twin studies, and genetic linkage analyses, support a genetic predisposition to PEX [2–5, 15, 16]. The underlying genetic mechanisms are thought to be due to the disruption of regulatory genes that are involved in both the production and the breakdown of extracellular material in PEX. The results of several studies suggest that the cross-linking enzyme lysyl oxidase-like 1 (LOXL1) participates in the stabilization of newly synthesized elastic proteins and finally in the stable accumulation of this material [17–19]. Clusterin (CLU) has been supposed to potentially influence the manifestation of the PEX syndrome [20–22]. Clusterin is a multifunctional protein which plays a role in many cellular processes ranging from lipid transport, acting as extracellular chaperone, to cellular proliferation and death and was found in most tissues and body liquids. The gene encoding this protein is induced by heat and oxidative and mechanical stress. It was found to be present in exfoliation deposits on anterior lens capsules, but the iris is the tissue where CLU was the most abundantly expressed gene [23, 24]. Studies on clusterin have also indicated that its deficiency may result in PEX material accumulation [25]. In PEX eyes, a significant downregulation of clusterin mRNA was seen in all anterior segment tissues, when compared to normal eyes. Clusterin aqueous humour levels were also significantly reduced in PEX eyes [26]. The clusterin presence in PEX deposits and reduced amounts in aqueous humour of PEX patients led to an investigation of the genetic variants of the CLU gene and its association with PEX syndrome. Nine SNPs of the CLU gene in 86 cases of PEX and 2422 controls from the Blue Mountains Eye Study Cohort were genotyped by Burdon et al. [27]. They found that variants of CLU gene do not strongly modify the risk of PEX in this population, but one SNP (rs3087554) haplotype with a frequency of 7% may confer some increased risk. The significant age difference between cases and controls makes the power of this study lower; the mean age of cases was six years greater than that of the controls. When the age of controls was restricted to 73 years or older, the association between SNP rs3087554 and PEX was not found (*p*=0.072). Krumbiegel et al. observed the association between PEX and SNP rs2279590 in intron 8 of the CLU gene in two German cohorts (*p*=0.0347, *p*=0.0244) [28]. This association was not confirmed in Italian patients (*p*=0.7173). None of the other evaluated SNPs of CLU were associated with PEX in both populations. The results of Burdon et al. and Krumbiegel et al. indicate that common genetic variation of *CLU* is not a strong genetic modifier of the risk of PEX, but may confer some increased risk in some populations [27, 28]. Padhy et al. revealed a genetic association between CLU SNP rs2279590 and PEX in Indian population with a *p* value of 0.004 [29]. The high risk allele “G” at rs2279590 has an effect on clusterin mRNA expression. There was a two-fold higher clusterin mRNA level in “GG” genotyped individuals in comparison to “AA” genotyped individuals (*p*=0.039). Five CLU SNPs (rs11136000, rs2279590, rs9331888, rs9331931, and rs3087554) were evaluated in the study of Dubey et al. [30]. These authors did not find any significant differences in the distributions of genotype and allele frequencies between PEX patients and control subjects in Indian population. The CLU SNP rs2279590 was evaluated in both aforementioned studies and their results were contradictory.

In our study, the possible association between two CLU SNPs, rs3087554 and rs2279590, and PEX syndrome in Polish population was assessed. There were no significant differences in the distributions of genotype and allele frequencies between PEX patients and controls. However, the distribution of haplotype frequencies differs between groups with *p* value equal 0.03. The haplotype (CT) was more common in control group than in PEX patients (*p*=0.0484), conferring an 18-fold decreased risk to the disease. The risk of patients with this haplotype (CT) for developing PEX is equal 0.0545. Due to the small groups studied and the fact that haplotype (CT) was not found at all in the PEX group, the results should be quantified with caution. The haplotype (TT) almost doubles the risk of PEX, but this result was not statistically significant (*p*=0.0687).

## 5. Conclusions

Our results indicate that CLU variants may contribute to the risk of PEX in Polish population.

## Figures and Tables

**Table 1:** Allele frequencies of rs3087554 polymorphisms in Polish population.

Allele	PEX patients		Controls		*p*
	*n*=162	Frequency	*n*=182	Frequency	
C	46	0.28	51	0.28	0.9388
T	116	0.72	131	0.72	

**Table 2:** Genotype frequencies of rs3087554 polymorphisms in Polish population.

Genotype	PEX patients		Controls		*p*
	*n*=81	Frequency	*n*=91	Frequency	
CC	7	0.09	6	0.07	0.8315
CT	32	0.40	39	0.43	
TT	42	0.52	46	0.51	

**Table 3:** Allele frequencies of rs2279590 polymorphisms in Polish population.

Allele	PEX patients		Controls	
	*n*=134	Frequency	*n*=100	Frequency
C	86	0.64	68	0.68
T	48	0.36	32	0.32

**Table 4:** Genotype frequencies of rs2279590 polymorphisms in Polish population.

Genotype	PEX patients		Controls	
	*n*=67	Frequency	*n*=50	Frequency
CC	29	0.43	21	0.42
CT	28	0.42	26	0.52
TT	10	0.15	3	0.06

**Table 5:** Frequencies of *CLU* haplotypes in PEX patients and controls.

Haplotype^*∗*^	Estimated haplotype frequencies (%)		*p* value (test Χ^2^)	OR (95% CI)	*p* value
	PEX patients (*n*=130)	Controls (*n*=98)			
TT	48 (37)	25 (26)	**0.0300**	1.7093 (0.9597–3.0443)	0.0687
TC	45 (35)	43 (44)		0.6772 (0.3953–1.1600)	0.1557
CC	37 (28)	24 (24)		1.2267 (0.6749–2.2298)	0.5028
CT	0 (0)	6 (6)		0.0545 (0.0030–0.9799)	**0.0484**

^*∗*^Order of the alleles is as follows: rs308755 and rs2279590.

## Data Availability

The data used to support the findings of this study are available from the corresponding author upon request.
